# Genome Sequences of *Listeria monocytogenes* Serotype 4b Variant Strains Isolated from Clinical and Environmental Sources

**DOI:** 10.1128/genomeA.00771-13

**Published:** 2013-10-17

**Authors:** Pongpan Laksanalamai, Susan R. Steyert, Laurel S. Burall, Atin R. Datta

**Affiliations:** Center for Food Safety and Applied Nutrition, Food and Drug Administration, Laurel, Maryland, USA

## Abstract

*Listeria monocytogenes* strains that show a novel PCR serotyping profile (IVb-v1) have been reported recently. Here, we announce the draft genome sequences of five *L. monocytogenes* IVb-v1 strains isolated from the United States and Australia that harbor a 6.3-kb DNA cassette characteristic of serotype 1/2a strains.

## GENOME ANNOUNCEMENT

*Listeria monocytogenes* is a Gram-positive food-borne pathogen that causes listeriosis, with 20 to 30% mortality and >95% hospitalization rates. The annual incidence of food-borne listeriosis in the United States is about 1,600 cases with 255 deaths (1). Based on somatic and flagellar antigens, *L. monocytogenes* strains can be grouped into 13 serotypes (2), of which serotypes 1/2a, 1/2b, and 4b represent the vast majority of the disease-causing strains (3–5). Due to the complexity involved in classical serotypic determination, a PCR-based method that targets four genes (*orf2110*, *orf2819, lmo01118*, and *lmo0737*) has been developed (6). Using this method, several 4b strains with an unusual PCR amplicon pattern, termed IVb-v1, have been reported (7–10). In addition, these IVb-v1 strains harbor a 6.3-kb DNA cassette that is characteristic of 1/2a strains (8).

To identify other genomic footprints of these IVb-v1 strains, we report the draft genome sequences of five *L. monocytogenes* serotype IVb-v1 strains isolated from two different continents. *L. monocytogenes* LS542 is an environmental isolate from a soft-cheese manufacturing facility in the United States, which was collected as part of monitoring of ready-to-eat food facilities by the FDA (9). Four clinical isolates were isolated from listeriosis patients in New South Wales (*L. monocytogenes* LS642, LS643, and LS644) and Victoria (*L. monocytogenes* LS645), Australia, in 2009. No epidemiological link has been established among the four Australian cases (10). Whole-genomic DNA was extracted using the Qiagen DNeasy blood and tissue kit (Qiagen, Valencia, CA), according to the manufacturer’s protocol with a slight modification (11). The library was prepared from the extracted genomic DNA using a Nextera sample preparation kit (Illumina, San Diego, CA), and a 2 × 250 paired-end sequencing run was performed on an Illumina MiSeq platform (12). The reads were trimmed and *de novo* assembled using the CLC Genomics Workbench version 6.0.5 (CLC bio, Germantown, MD). The resulting assemblies have >50× coverage and generated 45, 24, 30, 32, and 24 contigs representing the genomes of *L. monocytogenes* strains LS542, LS642, LS643, LS644, and LS645, respectively. The estimated genome size is ~2.9 Mb with a G+C content of 37.9% for each strain. BLAST analysis of these contigs against the 6.3-kb gene cassette (*lmo0733* to *lmo0740*) of *L. monocytogenes* EGD-e confirmed the existence of the gene cassette in all five IVb-v1 strains. The genome annotation, performed on the Rapid Annotations using Subsystems Technology (RAST) server (13), revealed that the genomes contain 2,899, 2,927, 2,830, 2,826, and 2,827 protein-coding genes for strains LS542, LS642, LS643, LS644, and LS645, respectively. The availability of these *L. monocytogenes* IVb-v1 genome sequences will facilitate in-depth study of these strains that may lead to the discovery of previously unidentified genes and help in understanding the significance and evolution of this group of strains.

### Nucleotide sequence accession numbers.

This whole-genome shotgun project has been deposited at DDBJ/EMBL/GenBank under accession no. AVQQ00000000, AVQM00000000, AVQN00000000, AVQO00000000, and AVQP00000000 for *L. monocytogenes* serotype IVb-v1 strains LS542, LS642, LS643, LS644, and LS645, respectively.
